# Metaplot: A new Stata module for assessing heterogeneity in a meta-analysis

**DOI:** 10.1371/journal.pone.0253341

**Published:** 2021-06-28

**Authors:** Jalal Poorolajal, Shahla Noornejad

**Affiliations:** 1 Department of Epidemiology, School of Public Health, Hamadan University of Medical Sciences, Hamadan, Iran; 2 Modeling of Noncommunicable Diseases Research Center, School of Public Health, Hamadan University of Medical Sciences, Hamadan, Iran; 3 School of Public Health, Hamadan University of Medical Sciences, Hamadan, Iran; Tabriz University of Medical Sciences, ISLAMIC REPUBLIC OF IRAN

## Abstract

**Background:**

The proposed sequential and combinatorial algorithm, suggested as a standard tool for assessing, exploring, and reporting heterogeneity in the meta-analysis, is useful but time-consuming particularly when the number of included studies is large. Metaplot is a novel graphical approach that facilitates performing sensitivity analysis to distinguish the source of substantial heterogeneity across studies with ease and speed.

**Method:**

Metaplot is a Stata module based on Stata’s commands, known informally as "ado". Metaplot presents a two-way (x, y) plot in which the x-axis represents the study codes and the y-axis represents the values of I^2^ statistics excluding one study at a time (n-1 studies). Metaplot also produces a table in the ’Results window’ of the Stata software including details such as I^2^ and χ^2^ statistics and their *P*-values omitting one study in each turn.

**Results:**

Metaplot allows rapid identification of studies that have a disproportionate impact on heterogeneity across studies, and communicates to what extent omission of that study may reduce the overall heterogeneity based on the I^2^ and χ^2^ statistics. Metaplot has no limitations regarding the number of studies or types of outcome data (binomial or continuous data).

**Conclusions:**

Metaplot is a simple graphical approach that gives a quick and easy identification of the studies having substantial influences on overall heterogeneity at a glance.

## Introduction

The studies that are brought together in a meta-analysis inevitably differ in many aspects. This variability across studies is called heterogeneity [1]. The between-studies heterogeneity can be assessed by the chi-square test also written as χ^2^ or Chi^2^ and can be quantified by I^2^ statistics [2, 3]. When there is heterogeneity in a meta-analysis, the source of heterogeneity across studies should be carefully investigated on a case-by-case basis [4].

A common approach, which was proposed by Patsopoulos et al, is to perform a sensitivity analysis based on a sequential and combinatorial algorithm [5]. According to this algorithm, one study is excluded from the meta-analysis at a time and the impact of the excluded study on the between-study heterogeneity is evaluated based on I^2^ statistic and χ^2^ test. This ‘one-out’ sensitivity analysis tells us to what extent the overall heterogeneity changes by excluding a particular study at a time. Then, the study that is responsible for the largest decrease in I^2^ value should be dropped out. This process is repeated for a new set of n-1 studies. This sequential and combinatorial algorithm is repeated several times until the I^2^ statistic drops below the desired threshold value of 50%. In the last step, there is a possibility that more than one omitted study can result in I^2^ dropping below the intended threshold. In such cases, the algorithm that results in the maximum decrease in the I^2^ statistic below the desired threshold is selected. There is a chance that two or more studies cause the same reduction in I^2^ by their exclusion. In this case, the study with the largest reduction in χ^2^ statistic (the least χ^2^ statistic) is dropped out.

Based on the aforementioned algorithm, this ‘one-out’ sensitivity analysis must be repeated n-1 times to specify and exclude the outlying study from the meta-analysis. If the desired threshold value of 50% is not achieved in the first step, the algorithm must be repeated n-2, n-3, etc. Therefore, this algorithm may be boring and time-consuming when the number of included studies is large and the between-studies heterogeneity is substantial.

In this study, we aimed to introduce a novel Stata graph that performs the ‘one-out’ sensitivity analysis for n-1 studies and identifies immediately the studies responsible for substantial heterogeneity across studies by executing "metaplot.ado" Stata command.

## Methods

Metaplot is a Stata module based on Stata’s commands, known as "ado". Metaplot produces a two-dimensional (x, y) Stata graph. The x-axis represents the included studies. The studies are shown on this axis by an ID code. The y-axis represents the values of I^2^ statistics based on ‘one-out’ (n-1 studies) sensitivity analysis indicating to what extent the overall heterogeneity changes by excluding a particular study at a time.

Furthermore, the "metaplot" command generates a table in the “Results window” of the Stata including more details about ‘one-out’ sensitivity analysis in terms of the I^2^ and χ^2^ statistics and their *P*-values. In addition to study codes, the studies’ identifications can be presented in the table.

The "metaplot" command is flexible and works with any measurement option including binary data (effect size + standard error or effect size + confidence intervals) and continuous data (sample + mean + standard deviation). The full form of the "metaplot" command is as follows

metaplot varlist [if] [in] [, id(study) tr(#)]

where

- “varlist” can be “a b c d” or “lnes se” or “es lles ules” or “n1 mean1 sd1 n0 mean0 sd0”
- “id(study)” option displays studies identifications (the first authors and the year of publication) specified by the variable “study” in the dataset.
- “tr(#)” option specifies the desired threshold values for example: 0.4, 0.5, 0.6, 0.65, 0.8, etc.

The abbreviations in the above command represent the following terms.

- “a b c d” represents “events” and “non-events” in the intervention (exposure) and control groups, respectively.
- “lnes” represents the “Naperian logarithm” of the effect size that may be risk ratio (lnrr) or odds ratio (lnor).
- “se” represents the standard error of the effect size.
- “es” represents the effect size that may be risk ratio (rr) or odds ratio (or).
- “lles” represents the lower limit of the confidence interval for the effect size.
- “ules” represents the upper limit of the confidence interval for the effect size.
- “n1” and “n0” represent the sample size for the intervention (exposure) and control groups, respectively.
- “mean1” and “mean0” represent the mean for the intervention (exposure) and control groups, respectively.
- “sd1” and “sd0” represent the standard deviation for the intervention (exposure) and control groups, respectively.

The relevant files including “metaplot.ado” and “metaplot.hlp” are attached to this paper as (S1 and S2 Files).

## Results

To show the capability and flexibility of the ’metaplot" command we used various datasets (S1–S3 Datasets) related to our previous published meta-analyses [6–8].

The first dataset (S1 Dataset), which was used to introduce the "metaplot" module, related to a published meta-analysis addressed the risk factors for stomach cancer [6]. This is a dataset with a “binomial” outcome (stomach cancer). In this meta-analysis, 15 studies addressed the association between stomach cancer and drinking black tea. The heterogeneity across studies was high (I^2^ = 64.23%). To perform sensitivity analysis using the “metaplot” command for this dataset, we executed the following command in the Stata software.

- metaplot es lles ules, id(study)

**Fig 1:**
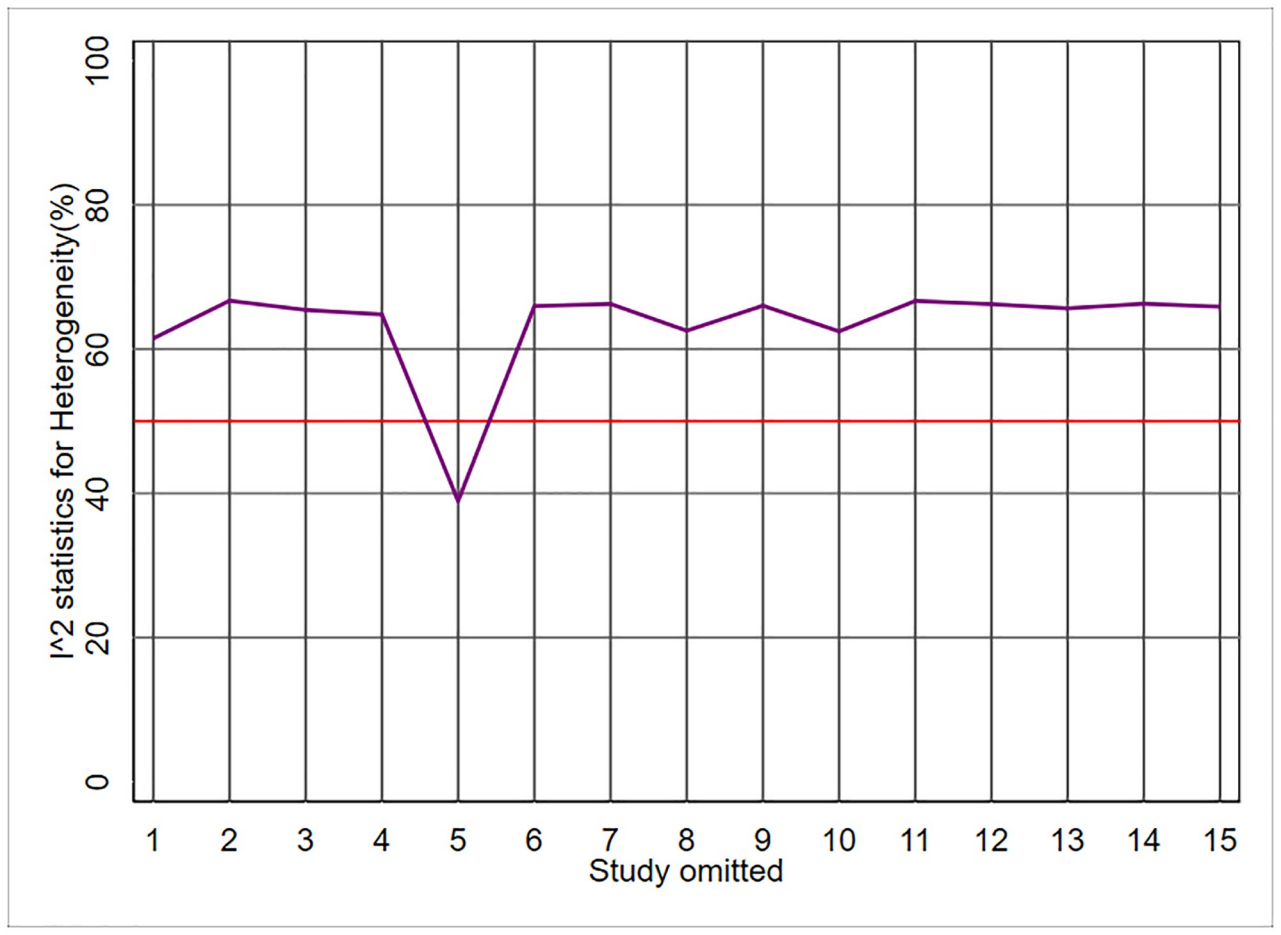
Meta-analyses of risk factors for stomach cancer; metaplot delineates I^2^ statistics and χ^2^ statistics and their *P*-values based on ‘one-out’ sensitivity analysis [Stata command: Metaplot es lles rules, id(study)].

The result of the above command is given in Fig 1. This figure shows the results of the ‘one-out’ sensitivity analysis using the "metaplot" command. According to this figure, all values of I^2^ statistics excluding one study at a time (n-1 studies) were above the desired threshold value of 50% except for study #5. By omitting study #5 from the meta-analysis, the heterogeneity fell below the desired threshold value of 50%. That means this study was an outlier and the main reason for heterogeneity across studies. Table 1 shows the results of ‘one-out’ sensitivity analysis in detail including I^2^ and χ^2^ statistics and their *P*-values omitting one study at a time. Based on this table, the overall heterogeneity across studies was high (I^2^ = 64.23%). However, the heterogeneity decreased to 38.93% after omitting study #5.

**Table 1:** Meta-analyses of risk factors for stomach cancer; results of "metaplot" command.

Study omitted	I2	[95% Conf. Interval]		Chi2	P>|t|
1 Baroudi 2014	61.48	31.09	78.46	33.75	0.001
2 Takezaki 2001	66.71	41.62	81.02	39.05	0.000
3 Goldbohm 1996	65.43	39.06	80.39	37.60	0.000
4 Gallus 2009	64.81	37.83	80.09	36.95	0.000
5 Chew 1999	38.93	0.00	67.60	21.29	0.067
6 Watabe 1998	65.98	40.16	80.66	38.21	0.000
7 Inoue 1994	66.26	40.72	80.80	38.53	0.000
8 Hoshiyama 1992	62.55	33.27	78.98	34.71	0.001
9 Al-qadasl 2016	66.03	40.25	80.68	38.26	0.000
10 Hansson 1993	62.46	33.08	78.94	34.63	0.001
11 Galanis 1998	66.68	41.57	81.01	39.02	0.000
12 Chen 2009	66.23	40.67	80.78	38.50	0.000
13 Inoue 1998	65.65	39.50	80.50	37.85	0.000
14 Bao 2004	66.29	40.78	80.81	38.57	0.000
15 La Vecchia 1992	65.88	39.96	80.61	38.10	0.000
**Combined**	**64.23**	**37.88**	**79.40**	**39.14**	**0.000**

The second dataset (S2 Dataset), which was used to introduce the "metaplot" module, related to a published meta-analysis addressed the effect of oral potassium supplementation on the management of essential hypertension [7]. This is a dataset with a “continuous” outcome (blood pressure). In this meta-analysis, 22 studies addressed the effect of oral potassium supplementation on diastolic blood pressure. The heterogeneity across studies was high (I^2^ = 81.88%). To perform sensitivity analysis using the “metaplot” command for this dataset, we executed the following command in the Stata software.

- metaplot n1 mean1 sd1 n0 mean0 sd0, id(study)

**Fig 2:**
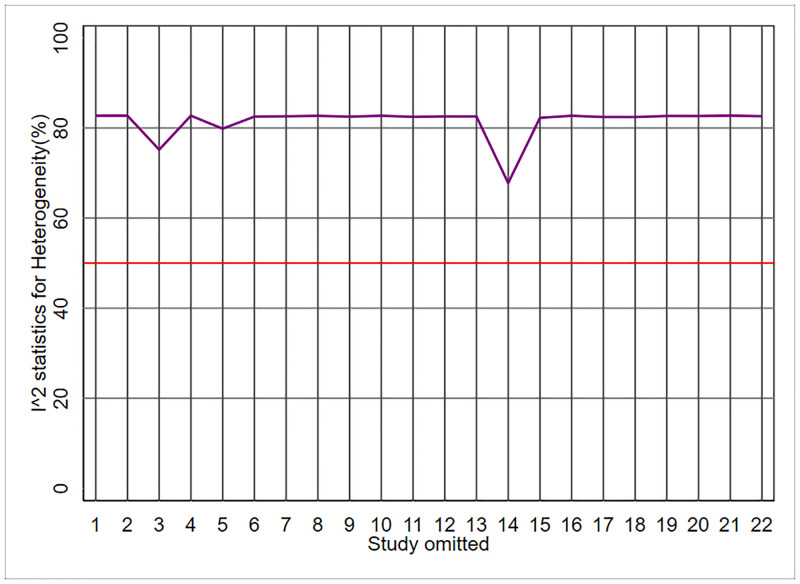
Meta-analyses of oral potassium supplementation for the management of essential hypertension; metaplot delineates I^2^ statistics and χ^2^ statistics and their *P*-values based on ‘one-out’ sensitivity analysis [Stata command: Metaplot n1 mean1 sd1 n0 mean0 sd0, id(study)].

The result of the above command is given in Fig 2. This figure shows the results of the "metaplot" command based on a ‘one-out’ sensitivity analysis. According to this figure, all values of I^2^ statistics excluding one study at a time (n-1 studies) were above the desired threshold value of 50%. However, the effect of omitting one study at a time was not similar across studies. For example, studies #14, #3, and #5 were responsible for the largest decrease in I^2^ values, respectively. Although heterogeneity decreased significantly, particularly by omitting study #14, it did not reach below the threshold value of 50%. Therefore, this process should be repeated for a new set of n-1 studies after omitting study #14. According to the results of Table 2, the overall heterogeneity across studies was high (I^2^ = 81.88%). However, the heterogeneity decreased to 67.76%, 75.19%, and 79.85% after omitting studies #14, #3, and #5, respectively.

**Table 2:** Meta-analyses of oral potassium supplementation for the management of essential hypertension; results of "metaplot" command.

Study omitted	I2	[95% Conf. Interval]		Chi2	P>|t|
1 Forrester 1988	82.72	74.64	88.23	115.74	0.000
2 Fotherby 1992	82.73	74.66	88.23	115.83	0.000
3 Franzoni 2005	75.19	62.10	83.75	80.60	0.000
4 Gijsbers 2015	82.73	74.65	88.23	115.79	0.000
5 Grimm 1988	79.85	69.93	86.49	99.24	0.000
6 Grobbee 1987	82.53	74.33	88.11	114.47	0.000
7 He 2010	82.60	74.44	88.15	114.92	0.000
8 Heseltine 1990	82.70	74.61	88.21	115.61	0.000
9 Kaplan 1985	82.51	74.30	88.10	114.37	0.000
10 Kawano 1998	82.72	74.64	88.23	115.75	0.000
11 Lawton 1990	82.47	74.23	88.07	114.09	0.000
12 MacGregor 1982	82.56	74.38	88.13	114.67	0.000
13 MacGregor 1984	82.56	74.38	88.13	114.67	0.000
14 Patki 199076	67.76	49.27	79.51	62.04	0.000
15 Rahimi 2007	82.25	73.88	87.94	112.71	0.000
16 Richards 1984	82.71	74.63	88.22	115.70	0.000
17 Siani 1987	82.43	74.17	88.05	113.82	0.000
18 Siani 1991	82.43	74.16	88.05	113.80	0.000
19 Smith 1985	82.67	74.56	88.19	115.40	0.000
20 Svetkey 1987	82.66	74.54	88.19	115.32	0.000
21 Valdes 1991	82.74	74.67	88.24	115.87	0.000
22 Wu 200682	82.62	74.47	88.16	115.05	0.000
**Combined**	81.88	73.51	87.6	115.88	**0.000**

The third dataset (S3 Dataset), which was used to introduce the "metaplot" module, related to a published meta-analysis addressed the preventable factors for primary prevention of childhood obesity [8]. This is a dataset with a “binomial” outcome (stomach cancer) and multiple studies. In this meta-analysis, 84 studies addressed the association between physical activity and childhood obesity. The heterogeneity across studies was high (I^2^ = 96%). We used the sequential and combinatorial algorithm and performed a ‘one-out’ sensitivity analysis and repeated the process several times. For this purpose, we executed the following command in the Stata software for n-1 studies several times.

- metaplot lnor se, id(study)

**Fig 3:**
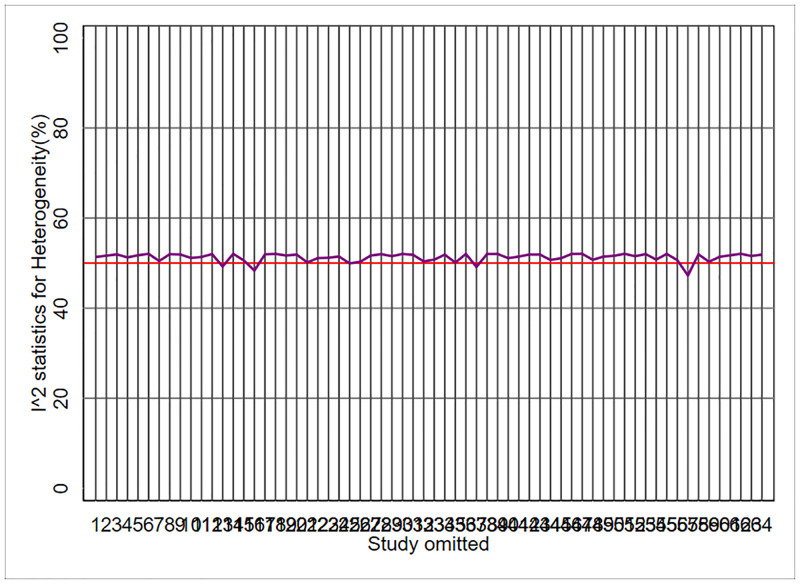
Meta-analyses of primary prevention of childhood overweight and obesity by preventable behavioral factors; metaplot delineates I^2^ statistics and χ^2^ statistics and their *P*-values based on ‘one-out’ sensitivity analysis [Stata command: Metaplot lnor se, id(study)].

The result of the above command is given in Fig 3. This figure shows the last step when the I^2^ statistic dropped below the desired threshold value of 50% by omitting just one more study. By looking at Fig 3 one can realize that there are at least 5 options to reduce the I^2^ statistic below the value of 50%. By omitting any of the studies #13, #16, #25, #37, and #57 the I^2^ statistic drops below the value of 50% and reaches 49.25%, 48.35%, 49.95%, 49.16%, and 47.25%, respectively (Table 3). When there is a possibility that more than one omitted study can result in I^2^ dropping below the intended threshold, the study that results in the maximum decrease in the I^2^ statistic below the desired threshold is selected. Accordingly omitting study #57 is the best choice. There might have been a chance that two or more studies caused the same reduction in I^2^ by their exclusion. In that case, the study with the largest reduction in χ^2^ statistic (the least χ^2^ statistic) would have been dropped out.

**Table 3:** Meta-analyses of primary prevention of childhood overweight and obesity by preventable behavioral factors; results of "metaplot" command.

Study omitted	I2	[95% Conf. Interval]		Chi2	P>t
1 Adachi-Mejia 2007	51.36	35.01	63.59	127.46	0.000
2 Al-Domi 2019	51.65	35.43	63.79	128.22	0.000
3 Al-Hazzaa 2012	51.93	35.83	63.99	128.98	0.000
4 Al-Muhaimeed 2015	51.27	34.88	63.53	127.23	0.000
5 Arango 2011	51.74	35.56	63.86	128.47	0.000
6 Badr 2017	52.06	36.01	64.08	129.32	0.000
7 Basterfield 2014	50.47	33.74	62.98	125.17	0.000
8 Bhuiyan 2013	51.97	35.89	64.02	129.09	0.000
9 Bibiloni 2010	51.91	35.80	63.97	128.92	0.000
10 Boričić 2014	51.15	34.71	63.45	126.92	0.000
11 De Lucinéia 2014	51.36	35.02	63.60	127.48	0.000
12 Dudas 2008	52.00	35.93	64.03	129.15	0.000
13 Duncan 2011	49.25	31.99	62.13	122.16	0.000
14 Dupuy 2011	52.06	36.02	64.08	129.33	0.000
15 Eker 2018	50.59	33.91	63.06	125.48	0.000
16 Fu 2004	48.35	30.70	61.51	120.04	0.000
17 Gharib 2008	51.94	35.85	64.00	129.01	0.000
18 Ghosh 2015	52.04	35.99	64.07	129.28	0.000
19 Godakanda 2018	51.69	35.49	63.82	128.34	0.000
20 Ha 2005	51.88	35.77	63.96	128.86	0.000
21 Hajian-Tilaki 2012	50.15	33.28	62.76	124.38	0.000
22 Haug 2009	51.10	34.65	63.41	126.80	0.000
23 Honório 2014	51.19	34.77	63.47	127.01	0.000
24 Januszek-Trzciakowska 2014	51.46	35.16	63.67	127.74	0.000
25 Keane 2017	49.95	32.99	62.61	123.87	0.000
26 Kuhle 2010	50.28	33.47	62.84	124.70	0.000
27 Leatherdale 2013	51.68	35.47	63.81	128.31	0.000
28 Liu 2012	51.98	35.90	64.02	129.11	0.000
29 Lowry 2012	51.54	35.27	63.72	127.93	0.000
30 Lätt 2015	52.02	35.96	64.05	129.23	0.000
31 Macwana 2017	51.84	35.70	63.92	128.73	0.000
32 Mahfouz 2011	50.38	33.61	62.91	124.95	0.000
33 Mansoori 2018	50.75	34.15	63.17	125.90	0.000
34 Melkevik 2015	51.87	35.75	63.95	128.83	0.000
35 Muntaner-Mas 2017	50.12	33.23	62.73	124.29	0.000
36 Mushtaq 2011	52.04	35.98	64.06	129.26	0.000
37 Nasreddine 2014	49.16	31.86	62.07	121.95	0.000
38 Neutzling 2003	52.02	35.96	64.05	129.22	0.000
39 Oellingrath 2017	52.02	35.97	64.06	129.23	0.000
40 Oliveira 2017	51.10	34.64	63.41	126.79	0.000
41 Orgiles 2014	51.45	35.14	63.65	127.70	0.000
42 Ortega 2007	51.91	35.80	63.97	128.91	0.000
43 Panagiotakos 2008	51.90	35.79	63.97	128.90	0.000
44 Pati 2014	50.69	34.06	63.13	125.75	0.000
45 Peart 2011	51.08	34.61	63.40	126.73	0.000
46 Peltzer 2011	52.03	35.98	64.06	129.26	0.000
47 Pengpid 2018	52.06	36.02	64.08	129.34	0.000
48 Rani 2013	50.73	34.12	63.16	125.85	0.000
49 Rosi 2017	51.44	35.14	63.65	127.69	0.000
50 Saikia 2016	51.61	35.37	63.77	128.13	0.000
51 Savva 2002	52.06	36.02	64.08	129.33	0.000
52 Scanferla de Siqueira 2007	51.52	35.25	63.71	127.90	0.000
53 Shankaran 2011	52.00	35.93	64.04	129.16	0.000
54 Silva 2016	50.79	34.19	63.20	125.98	0.000
55 Silveira 2006	52.04	35.99	64.06	129.27	0.000
56 Teo 2014	50.64	33.99	63.10	125.62	0.000
57 Thibault 2010	47.25	29.11	60.75	117.54	0.000
58 Urrutia-Rojas 2008	51.94	35.85	64.00	129.02	0.000
59 Veugelers 2005	50.30	33.49	62.86	124.74	0.000
60 Watharkar 2015	51.41	35.08	63.63	127.59	0.000
61 Wethington 2013	51.72	35.53	63.84	128.42	0.000
62 Wilkie 2016	52.06	36.02	64.08	129.33	0.000
63 Winkvist 2016	51.58	35.33	63.74	128.04	0.000
64 Wittmeier 2008	51.89	35.77	63.96	128.86	0.000
**Combined**	**51.29**	**35.06**	**63.46**	**129.34**	**0.000**

## Discussion

The idea of Metaplot, which was first introduced in 2010 [9], is a simple graphical approach to identify outliers and their effects on overall heterogeneity across studies. Patsopoulos et al. [5] suggested the sequential and combinatorial algorithm for performing sensitivity analyses. This algorithm is a useful method for assessing, exploring, and reporting the between-study heterogeneity in the meta-analysis but is time-consuming when the number of included studies is large and heterogeneity is substantial. For example, as noted in the results section, 84 studies addressed the association between physical activity and childhood obesity [8]. In this case, the sequential and combinatorial algorithm needs to be repeated hundreds of times particularly when the heterogeneity across studies is substantial. While by executing the "metaplot" command we can perform ‘one-out’ sensitivity analysis across several studies, no matter how many they are, and identify immediately to what extent the overall heterogeneity changes by excluding a particular study at a time. Another capability of the "metaplot" command is its flexibility. It is possible to execute this command for meta-analysis of different types of outcome data (e.g. binary, continuous, or time to event) and different types of summary measures (e.g. odds ratio, risk ratio, rate ratio, or hazard ratio).

The I^2^ threshold value of 50% usually depends on the type of research we are performing. The threshold value of 50% is not rigid in the "metaplot" command. A rigid threshold value for the interpretation of I^2^ can be misleading since the importance of inconsistency depends on several factors [1]. The "metaplot" command has the option "tr(#)" that establishes different threshold values.

Care must be taken in the interpretation of the chi-squared test since it has low power in the situation of a meta-analysis when studies have a small sample size or are few in number. This means that while a statistically significant result may indicate a problem with heterogeneity, a non-significant result must not be taken as evidence of no heterogeneity [1]. This is also why a P-value of 0.10 is sometimes used, rather than the conventional level of 0.05. Another problem with the test is that when there are many studies in a meta-analysis, the test has a high power to detect a small amount of heterogeneity that may be clinically unimportant.

Huedo-Medina et al. [10] examined and compared the performances of the Q test and the I^2^ index for assessing homogeneity across individual studies in meta-analysis. They confirmed that the Q test only reports the presence or absence of homogeneity across studies but does not specify the extent of such heterogeneity. On the other hand, the I^2^ index can quantify the degree of heterogeneity. Although the I^2^ index has the same problems of low statistical power with a small number of studies, they suggested the I^2^ index as a complement to the Q test.

The raw idea of “metaplot” was first introduced in 2010 [9]. This preliminary idea was never implemented actually at that time because the package had not been generated yet. The new design of the “metaplot” presented in this paper is very different from the original one introduced in 2010. The original design was a complicated three-dimensional graph with x, y, and z axes including unnecessary information. It was rather hard to understand. The new design of “metaplot” is a two-dimensional graph with x and y axes. Furthermore, we added a table including details of information (I^2^ and χ^2^ statistics and their *P*-values omitting one study in each turn) to simplify the interpretation of the ‘metaplot’ graph. In the current paper, we explained the capability of the “Metaplot” module and how to use the Stata command and its options. We examined this module on different real datasets and reported the results.

There are several graphical methods for the exploration of heterogeneity in the meta-analysis. One of these methods is the traditional Galbraith plot [11, 12]. This plot provides a graphical display to get a visual impression of the amount of heterogeneity from a meta-analysis. For each study, the observed effect sizes on the vertical axis are plotted against the reciprocal standard errors on the horizontal axis. The regression line projects through the origin, with its 95% confidence interval positioned 2 units over and below the regression line, has a slope equal to the overall log rate ratio. In the absence of heterogeneity, we could expect all the points to lie within the confidence bounds. The L’Abbé plot is another useful method for assessing heterogeneity in the meta-analysis [13, 14]. It is a scatter plot with the risk in the control group on the x-axis and the risk in the experimental group on the y-axis. The visual inspection gives a quick and easy indication of the studies having different results from other studies. These studies are considered outliers and hence potential sources of heterogeneity. Although these graphical procedures are useful and their interpretations are straightforward, they have a major limitation. When only one study causes extreme heterogeneity, these methods point to the same study as Metaplot suggests. However, in situations where the heterogeneity is resulted from several studies, the above graphical procedures are impractical to indicate to what extent a particular study influences the overall heterogeneity. Our proposed graphical method has overcome this problem. According to Metaplot method, one study is excluded from the meta-analysis at a time and the impact of the excluded study is evaluated on the overall heterogeneity. This ‘one-out’ approach tells us to what extent the overall heterogeneity changes by excluding a particular study at a time.

The Metaplot has a limitation. When the number of studies is very large (more than 35) as shown in Fig 3, the study codes in the x-axis come together and even may collapse due to space constraints. In such cases, the identification of the study codes may be difficult. Fortunately, the properties of the “metaplot” module solved this problem. In addition to the "Metaplot", this module generates a table in the “Results window” of the Stata and gives more details of ‘one-out’ sensitivity analysis including the I^2^ and the χ^2^ statistics and their *P*-values as well as the studies codes and the studies identifications. Therefore, by turning back to the “Results window” we can realize which study has the greatest impact on the overall heterogeneity based on the I^2^ and χ^2^ statistics.

## Conclusion

Metaplot is a visual complementary approach for testing between-study heterogeneity. This plot is a simple graphical approach that gives a quick and easy identification of the studies having substantial influences on overall heterogeneity as fast as possible. This method is based on ‘one-out’ sensitivity analysis and provides information both graphically and quantitatively about the extent of the overall heterogeneity changes by excluding a particular study at a time in terms of I^2^ and χ^2^ statistics. It is possible to implement this graph for the meta-analysis of different types of outcome data.

## Supporting information

S1 File (ADO)

S2 File (HLP)

S1 Dataset (DTA)

S2 Dataset (DTA)

S3 Dataset (DTA)
