# Supplementary material for: Metaplot: A new Stata module for assessing heterogeneity in a meta-analysis
Source: PLoS One. 2021 Jun 28;16(6):e0253341. doi: 10.1371/journal.pone.0253341 (PMC8238175; doi:10.1371/journal.pone.0253341)
Supplement: Attachment — Submitted filename: Poorolajal et al - reviewer comments.pdf [file pone.0253341.s006.pdf]

Reviewer comments: "Metaplot: A new Stata module for assessing heterogeneity in a meta-analysis"

Sections quoted from the manuscript are in red.

2021-05-07

- Title
  - Is this actually a new Stata module or an update of the older Metaplot module? If it is the latter, language should be changed accordingly.
- Abstract
  - Results: first sentence presupposes that there is one study causing heterogeneity when that may or may not be the case. Consider instead: *Metaplot allows rapid identification of studies that have a disproportionate impact on heterogeneity across studies, and communicates to what extent omission of that study may reduce the overall heterogeneity based on the I2 and χ2 statistics.*
  - Results: I'm not sure these are so much results as an assertion as to what the authors believe Metaplot can do. It might be better to overview the performance of Metaplot in the practical examples here.
- Introduction
  - Your opening sentence is made a bit awkward by the inclusion of a non-defining clause where a defining clause should be used. Easily fixed by changing it to: *The studies that are brought together in a meta-analysis inevitably differ in many aspects.*
- Methods
  - Potential typo at the bottom of page 3. Language is referred to as "Meta" but functions are called "Mata" functions. I'm not a Stata user so please ignore this comment if my understanding is incorrect.
  - Is the first paragraph introducing mata functions even necessary given that you don't use or discuss this information later in the paper?
- Discussion
  - Performing sensitivity analyses based on the sequential and combinatorial algorithm proposed by Patsopoulos et al [5]. This is a sentence fragment and should be corrected.
  - "boring," subjective judgement. "Time consuming" is sufficient. Consider exclusion.
  - Although "metaplot" was first introduced in 2010[10], however, it was a preliminary idea that changed a lot over time. "However" can be removed here.
  - Although "metaplot" was first introduced in 2010[10], however, it was a preliminary idea that changed a lot over time. The new design of the "metaplot" presented in this paper is very different from the original one introduced in 2010. The original design was a complicated three-dimensional graph with x, y, and z axes including unnecessary information. It was rather hard to understand. The new design of "metaplot" is a two-dimensional graph with x and y axes. Furthermore, we added a table including details of information (I2 and χ2 statistics and their P-values omitting one study in each turn) to simplify the interpretation of the 'metaplot' graph.
    So what this paper is actually introducing is a modification to an existing package? If I'm understanding this paragraph correctly, the updated package is using fundamentally the same methodology but provides a changed graphical output and additional tables that

improve ease of interpretation. While I support providing peer reviewed documentation for statistical packages that can be cited in papers that use the package, I don't think that this manuscript introduces sufficient new information (or introduces old information in a sufficiently more comprehensive or accessible manner) to warrant a full research article. This feels like something that could be attached to the patch notes for the package.
